# In vitro functional analysis of lysyl oxidase family members in highly metastatic pancreatic cancer cells derived from a syngeneic orthotopic model

**DOI:** 10.1186/s12885-026-16173-1

**Published:** 2026-06-15

**Authors:** Kei Takahashi-Yamashiro, Kensuke Miyauchi, Kazuki Shimomura, Mizuki Nishikawa, Yasuyuki Morishita, Masako Nakanishi, Shinya Hayami, Manabu Kawai, Kohei Miyazono, Shogo Ehata

**Affiliations:** 1 Department of Molecular Pathology, Graduate School of Medicine, The University of Tokyo, 7-3-1 Hongo, Bunkyo-ku, Tokyo, 113-0033 Japan; 2 Department of Applied Pathology, Graduate School of Medicine, The University of Tokyo, 7-3-1 Hongo, Bunkyo-Ku, Tokyo, 113-0033 Japan; 3 Department of Chemistry, Faculty of Science, University of Alberta, 11227 Saskatchewan Drive, Edmonton, AB T6G 2G2 Canada; 4 Department of Pathology, School of Medicine, Wakayama Medical University, 811-1 Kimiidera, Wakayama-shi, Wakayama, 641-8509 Japan; 5 Second Department of Surgery, School of Medicine, Wakayama Medical University, 811-1 Kimiidera, Wakayama-shi, Wakayama, 641-8509 Japan; 6 Department of Human Pathology, School of Medicine, Wakayama Medical University, 811-1 Kimiidera, Wakayama-shi, Wakayama, 641-8509 Japan

**Keywords:** Pancreatic ductal adenocarcinoma, Metastasis, EMT, Lysyl oxidase family, Syngeneic orthotopic inoculation model

## Abstract

**Background:**

Pancreatic ductal adenocarcinoma (PDAC) is one of the highly lethal malignancies, with no established and effective cure. Therefore, identifying novel molecular targets for this cancer remains an urgent priority.

**Methods and results:**

We employed a syngeneic orthotopic inoculation model using murine pancreatic cancer Panc02 cells to better recapitulate the pancreatic tumor microenvironment. We established highly metastatic derivatives through three serial transplantation cycles, designated Panc02-3P cells. These cells exhibited enhanced tumorigenicity and metastatic potential both in vitro and in vivo. The RNA-sequence analysis revealed that Panc02-3P cells exhibited mesenchymal-like gene expression changes, compared with parental Panc02 cells, despite no significant changes in EMT-related transcription factor expression. In addition, we found that members of the lysyl oxidase (LOX) family, including LOX, LOXL1, and LOXL3, were markedly upregulated in Panc02-3P cells. The loss-of-function assays using siRNAs targeting LOX and LOXL1 demonstrated that the suppression of these genes significantly attenuated the migratory ability of Panc02-3P cells.

**Conclusion:**

Collectively, our findings suggest that the LOX family members are associated with the acquisition of a highly migratory and mesenchymal-like phenotype in metastatic pancreatic cancer cells and may contribute to tumor progression.

**Supplementary Information:**

The online version contains supplementary material available at 10.1186/s12885-026-16173-1.

## Background

Pancreatic ductal adenocarcinoma (PDAC) is one of the most aggressive malignancies, with a low 5-year survival rate of around 10% [1]. Although numerous efforts have been made to develop more effective therapeutic agents, only limited chemical options are available; surgical dissection is still the only curative treatment. Nearly half of patients with pancreatic cancer present with distant metastases, including liver metastasis, causing a poor prognosis. PDAC develops through a multi-step oncogenic process with sequential genetic alterations in key driver genes, including *KRAS*, *TP53* (p53), *CDKN2A/INK4A* (p16), and *SMAD4/DPC4* [2, 3]. More than 90% of PDACs harbor activating *KRAS* mutations, which constitutively activate phosphoinositide 3-kinase (PI3K)-AKT downstream pathways and induce subsequent cell cycle progression, survival, and invasion [2]. Inactivation of cyclin-dependent kinase (CDK) inhibitors, *CDKN2A* and *CDKN1A*, was observed in 90% of PDACs and in 20–80% even at an early pancreatic intraepithelial neoplasia (PanIN) state [2], contributing to tumor initiation of PDAC [2]. Moreover, alterations in *TP53* and *SMAD4* occur in 50–70% and 60–90% of advanced PDACs, respectively [2]. These sequential gene alterations collectively establish the malignant and heterogeneous nature of PDAC.

In addition to these gene alterations, the tumor microenvironment (TME) has been implicated in PDAC progression. PDAC is characterized by extensive fibrosis accompanied by an intense desmoplastic reaction [4, 5]. Pancreatic stellate cells produce abundant extracellular matrix (ECM) components within the fibrotic stroma through secretion of growth factors, including transforming growth factor-β (TGF-β) and platelet-derived growth factor (PDGF) [4, 6]. These observations indicate highly activated interactions between cancer cells and pancreatic TME. Mouse orthotopic inoculation models are widely used to investigate the functional roles of TME. Our group previously demonstrated the significance of the pancreatic microenvironment in PDAC progression using a human PDAC orthotopic xenograft model [7]. This model allowed us to expose cancer cells to pancreatic TME. However, that model was constructed by transplanting human pancreatic cancer cells into immunocompromised mice, limiting the physiological relevance of tumor–stroma interactions. In the present study, we orthotopically injected murine pancreatic cancer Panc02 cells into syngeneic C57BL/6 mice and established highly malignant derivatives under immunocompetent conditions.

Cancer cells acquire motility and invasiveness through multiple mechanisms, including epithelial-mesenchymal transition (EMT), resulting in local invasion, intravasation, and distant metastasis [8, 9]. During EMT, cancer cells lose their epithelial phenotype and simultaneously gain a mesenchymal phenotype. In addition, EMT contributes to cancer stem cell-like properties and chemoresistance. EMT is regulated by a set of transcription factors (EMT-TFs), including Snail, Slug, ZEB1/2, and Twist, which repress epithelial markers such as E-cadherin. In addition to EMT, extracellular matrix (ECM) remodeling plays a critical role in cancer progression and metastasis. The lysyl oxidase (LOX) family, consisting of LOX and LOX-like proteins (LOXL1–4), catalyzes the crosslinking of collagen and elastin, thereby regulating ECM structure and stiffness [10, 11]. Accumulating evidence suggests that LOX family members contribute to tumor progression by modulating the tumor microenvironment and enhancing cancer cell invasion and metastatic dissemination [12, 13]. Increased expression of LOX family members has been associated with poor prognosis in multiple cancer types [14, 15].

We characterized and identified the upregulation of LOX family members in highly malignant Panc02 derivatives established from a syngeneic orthotopic inoculation model. We further investigated the functions of LOX family members in PDAC progression, particularly their involvement in cancer cell migration and invasion.

## Methods

### Cell culture and reagents

Panc02 cells, derived from the tumors induced by 3-methyl-cholanthrene (3-MCA) [16], have a *Smad4* mutation, but no other mutations were observed (Supplementary Table 1) [17]. Panc02 cells (National Cancer Institute; Bethesda, MD) were maintained in RPMI 1640 (Thermo Fisher Scientific; Waltham, MA) supplemented with 10% fetal bovine serum (FBS, Thermo Fisher Scientific), penicillin, and streptomycin. Panc02 cells were infected with lentiviral vectors that introduce mCherry under the EF promoter using a lentiviral expression system (kindly provided by Prof. Hiroyuki Miyoshi, formerly Keio University, Tokyo, Japan) and used as parental Panc02 cells [18]. Cells were cultured in a 37 °C incubator with 5% CO_2_.

### Mouse pancreatic tumor model

All experiments were approved by and conducted according to the guidelines of the Animal Care and Use Committee of the Graduate School of Medicine, The University of Tokyo. C57BL/6 J mice (6 weeks, female) were purchased from CLEA Japan, Inc. (Tokyo, Japan). Panc02 cells were inoculated orthotopically into the pancreas under anesthesia using 29 G (5 × 10^5^ cells/50 μL) as previously described [7]. Mice were euthanized by isoflurane overdose (Fujifilm Wako Chemicals, Osaka, Japan), followed by cervical dislocation as a secondary method to confirm death. The experimental endpoint was set at 4 weeks after orthotopic inoculation and 2 weeks in the lung metastasis model. Mice were monitored regularly, and humane endpoints were applied when signs of significant emaciation were observed.

### Establishment of highly metastatic derivatives

Highly metastatic derivatives were established as previously described [7]. Primary tumor tissues in mice bearing parental Panc02 cells were excised and mechanically dissociated. The resulting cell suspension was cultured with RPMI 1640 medium supplemented with FBS, penicillin, and streptomycin. After 1–3 day culture, viable cells were attached on the bottom of the plate and the floating cells, including dead cells or lymphocytes, were removed by changing the culture medium. The adherent cells were passaged with 0.05% trypsin and ethylenediaminetetraacetic acid (EDTA). This process effectively removes fibroblasts through differential trypsinization. Following a couple of passages, highly purified populations of cancer cells were obtained. After purification, the cells were inoculated orthotopically into the pancreas of C57BL/6 J mice and allowed to progress. After three cycles of these processes, highly malignant derivatives were established, named Panc02-3P (3P#1 and 3P#2 were derived from different mice). Parental Panc02 cells or derivatives (Panc02-3P) were inoculated orthotopically (1–2 × 10^5^ cells/50 μL/mouse) to study tumorigenic ability in vivo. Mice were euthanized 4 weeks after inoculation, and primary tumors were excised. Cells were injected via the tail veins intravenously (1 × 10^5^ cells/mouse) and lungs were examined 2 weeks after injection to evaluate metastatic ability.

### Microscopic examination

Lungs were excised from mice intravenously injected with cancer cells and fixed in 4% paraformaldehyde (PFA) for at least 24 h. Fixed tissues were paraffin-embedded using an automated embedding system and sectioned with a microtome. Sections were deparaffinized in xylene, rehydrated through a graded ethanol series, and stained with hematoxylin and eosin (H&E). After washing, sections were dehydrated in graded ethanol, cleared in xylene, and mounted with Malinol. Histological evaluation was performed using an AX80 microscope (Olympus, Tokyo, Japan).

### Small interfering RNA (siRNA)

Stealth siRNAs targeting LOX and LOXL1 (siLOX s9288 and siLOXL1 s69292, respectively) and control siRNA (siNTC) were purchased from Thermo Fisher Scientific. Cultured cells were transduced with siLOX, siLOXL1, or siNTC at a final concentration of 10 nM using the Lipofectamine RNAiMAX Transfection Reagent (Thermo Fisher Scientific).

### RNA-sequence (RNA-seq) analysis

Cells were collected and processed as previously described [7]. Total RNA was extracted from the cells (parental Panc02, Panc02-3P#1, and Panc02-3P#2) using the RNeasy Mini Kit (Qiagen; Hilden, Germany), and genomic DNA was removed using RNase-Free DNase Set (Qiagen). Next, mRNA was isolated from the total RNA. With the Ion Total RNA-Seq Kit v2 (Thermo Fisher Scientific), mRNA fragmentation, adaptor accession, reverse transcription, amplification, and cDNA library establishment were performed using the Ion Total RNA-Seq Kit v2 (Thermo Fisher Scientific). Sequencing was conducted using the Ion Chef System and Ion Proton with the Ion PI IC 200 Kit. The expression of genes was calculated for the reads per kilobase of exon per million mapped sequence reads (RPKM). The data were applied to the gene set enrichment analysis (GSEA, https://www.gsea-msigdb.org/gsea/index.jsp) [19–21]. The RNA-seq analysis results were subjected to principal component analysis (PCA). As feature values, RPKM of genes that were more than three in at least one subline and whose coefficient of variation among Panc02 derivatives was more than 0.25 were selected. Next, the RPKM of these genes was normalized between 0 and 1. The lists of the top genes with high contributions in the first or second principal component were made with a threshold, where the sum of the squares of each gene’s contribution was 0.5. These lists were analyzed using DAVID’s Functional Annotation Clustering [22, 23]. Raw and processed data are available at GEO (GSE314975).

### Real-time PCR analysis

The cells were seeded into a 6-well plate (8 × 10^5^ cells/well) and cultured at 37 °C. On the following day, cells were harvested with Isogen (Nippon Gene; Toyama, Japan) to extract the total RNA. Next, the cDNA was synthesized by reverse transcription using the PrimeScript II 1 st strand cDNA synthesis kit (Takara Bio; Shiga, Japan). Gene expression was measured using the Fast SYBR Green Master Mix (Roche Diagnostics; Basel, Switzerland). The expression was normalized by that of hypoxanthine phosphoribosyltransferase 1 (*Hprt1*).

### Immunoblotting

Cells were seeded into a 12-well plate (2.5 × 10^5^ cells/well) and collected on the following day. Protein blotting was performed as previously described [7, 24]. Mouse anti-E-cadherin (1:1000, 610,181, BD, Franklin Lakes, NJ), mouse anti-α-tubulin (1:1000, T9026, Sigma-Aldrich, St Louis, MO) antibodies, and the matching secondary antibodies with horseradish peroxidase (HRP) were used.

### Cell proliferation assay

Cells were seeded into a 24-well plate (1 × 10^4^ cells/well) and cultured for 3 days at 37 °C. The cell number was counted using the Cell Count Reagent SF (Nacalai Tesque; Kyoto, Japan). The absorbance at 450 nm and 595 nm (control) was measured using a plate reader (Model 680 Microplate Reader; Bio-Rad, Hercules, CA).

### Transwell migration assay

The cells were seeded into an upper well of a transwell chamber (24-well plate, 3 × 10^3^ cells/well). After 24 h of incubation, the chambers were fixed with 4% PFA and stained with crystal violet or Giemsa. The areas of stained cells were measured using FIJI (https://fiji.sc/).

### Wound closure assay

The cells were seeded into a 24-well plate 1 day before the assay. The surface of the well was scratched with a pipette chip. After 48 h of incubation at 37 °C, the wound closure was checked under the microscope. The closure of the wound area was calculated by dividing the “average wound area after the 48 h incubation” by “average initial wound area” × 100 (%) using FIJI.

### Phalloidin staining

The cells were seeded into a chamber slide (8-well, Ninc Lab-Tek, Thermo Fisher Scientific, 2.5 × 10^4^ cells/well) and cultured overnight. On the following day, the cells were fixed with 4% PFA and permeabilized with 0.5% Triton X-100 in phosphate-buffered saline (PBS). After staining with phalloidin labeled with fluorescein isothiocyanate (FITC) (Sigma), slides were mounted with a mounting solution containing DAPI (Vector Laboratories; Newark, CA). The images were acquired using a microscope (BZ-X; KEYENCE, Osaka, Japan).

### Database analysis (survival)

The mRNA expression data (RNA Seq V2) were obtained from cBioPortal (TCGA Pancreatic Adenocarcinoma Firehose Legacy, *n* = 186). The Kaplan–Meier survival analysis was performed by classifying patient tumor samples based on LOXs expression, where a high expression was defined as EXP > 0.

### Statistical analysis

All statistical analyses were performed using the JMP Pro software version 14.1 (SAS Institute Japan, Tokyo, Japan). A one-sided Student’s *t*-test was used for comparison between two groups. The log-rank test was performed to evaluate differences in survival between two groups.

## Results

### Establishment of highly metastatic derivatives of pancreatic cancer cells in a syngeneic mouse tumor model

**Fig. 1:**
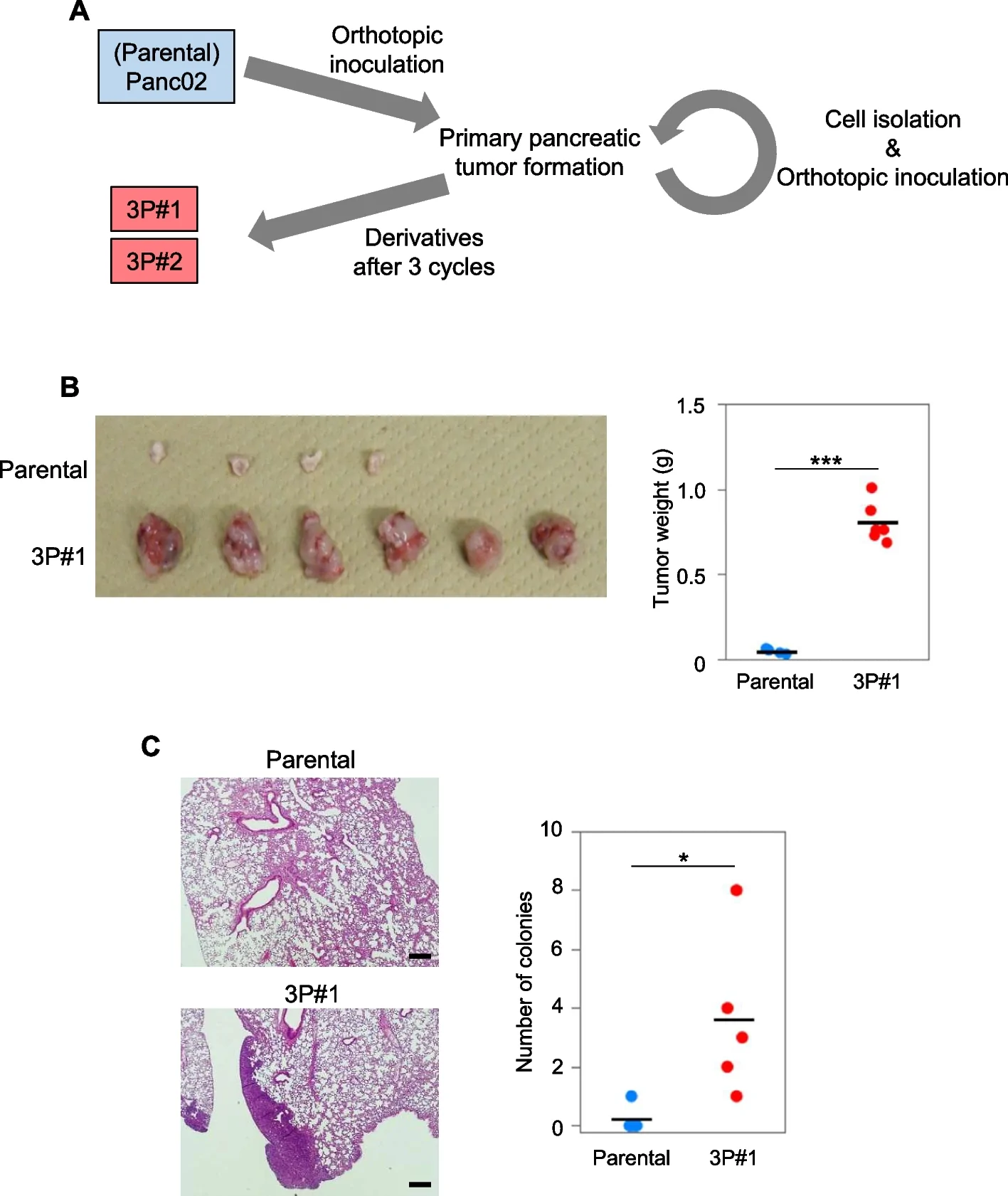
Establishment of highly malignant Panc02 derivatives using pancreatic orthotopic inoculation model. **A** Establishment of highly malignant Panc02 derivatives. Parental Panc02 cells were inoculated orthotopically into the pancreas of C57BL/6J mice. Four weeks after inoculation, primary tumors were excised, and cancer cells were isolated by culturing in vitro. Next, these isolated cancer cells were orthotopically inoculated into the pancreas of another mouse again. We repeated this process three times and established highly malignant derivatives named Panc02-3P (#1 and #2 derived from different mice). **B** Tumorigenic ability of established Panc02 derivatives in vivo. Parental Panc02 and derivative 3P#1 cells were inoculated orthotopically into the pancreas of C57BL/6J mice. Primary tumors were excised 20 days after inoculation (left). The tumor weight was measured after excision (right). Data are presented as mean ± SD (Parental; *n* = 4, 3P#1; *n* = 6). **C** Metastatic ability of Panc02 derivatives in vivo. Parental Panc02 and Panc02 derivative 3P#1 cells were injected intravenously in C57BL/6J mice (1 × 10^5^ cells/mouse). Two weeks after the injection, the lungs were excised and subjected to H&E staining (left). The number of metastatic colonies in one section was counted (right). Bar, 100 mm. Data are presented as mean ± SD (*n* = 5). * *P* < 0.05. *** *P* < 0.001

We obtained highly metastatic derivatives to elucidate the molecular mechanism underlying pancreatic cancer metastasis. We have already reported that the malignant characteristics of pancreatic cancer cells were gained by orthotopic inoculation, which recapitulates physical interactions between pancreatic cancer cells and pancreatic TME [7]. Panc02 (parental Panc02), mouse pancreatic cancer cells, were orthotopically injected into the pancreas in syngeneic C57BL/6 J mice. After three serial transplantations, derivatives were established from primary tumors as Panc02-3P#1 (3P#1) and Panc02-3P#2 (3P#2) (Fig. 1A). The tumorigenicity of 3P#1 cells was compared to that of parental cells using orthotopic inoculation. 3P#1 cells produced a larger primary tumor in vivo (Fig. 1B). Since primary tumor size differed markedly between these derivatives, metastatic potential was examined using an experimental lung metastasis model via intravenous injection to evaluate metastatic ability independent of primary tumor size. An increased number of metastatic colonies was observed in the lungs of mice injected with 3P#1 cells (Fig. 1C). Based on these results, we confirmed that Panc02-3P cells were defined as highly metastatic derivatives and used in the following studies.

### Characterization of highly metastatic derivatives Panc02-3P

**Fig. 2:**
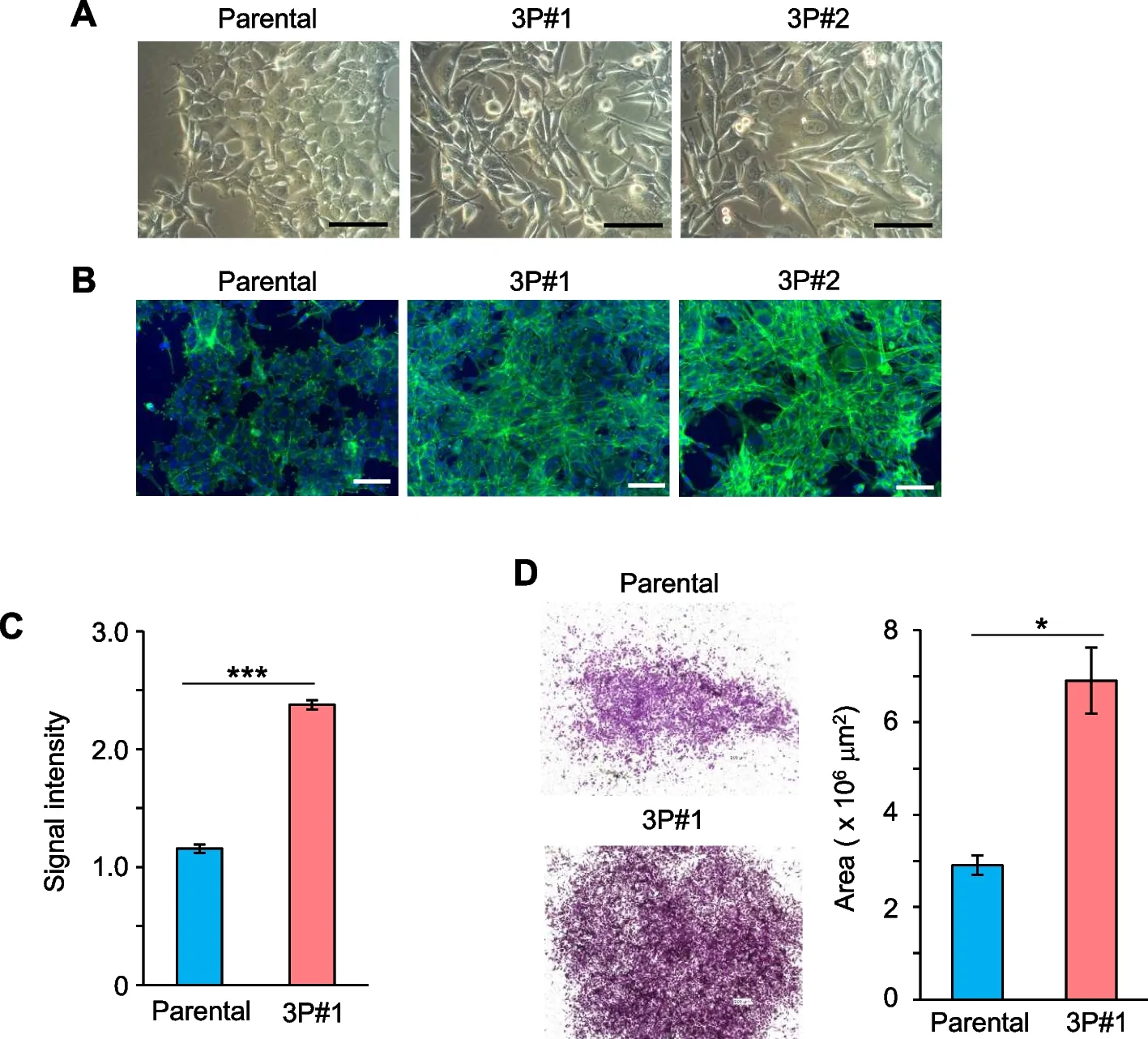
Characterization of Panc02 derivatives in vitro*.* **A** Cell morphology of Panc02 derivatives. The morphologies of parental Panc02 and Panc02-3P cells are shown. Bar, 100 μm. **B** F-actin in Panc02 derivatives. Phalloidin staining was performed with parental Panc02 and Panc02-3P cells (blue: DAPI, green: F-actin). Bar, 100 μm. **C** Cell proliferation ability of Panc02 derivatives in vitro. Proliferation assay was performed using the Cell Count Reagent SF with parental Panc02 and Panc02-3P#1 cells. The data at 3 days after incubation are shown. Data are presented as mean ± SD (*n* = 3). **D **Invasive ability of Panc02 derivatives in vitro. Transwell migration assay was conducted with parental Panc02 and Panc02-3P#1 cells. The chambers were collected after 24 h of incubation and migrated cells were stained with crystal violet. The images of migrated cells (left) and the areas of migrated cells in central view (right) are shown. Data are presented as mean ± SD (*n* = 3). * P < 0.05. *** P < 0.001

Multiple cellular phenotypes are involved in the metastatic process of cancer cells. Cellular phenotypes that are relevant to enhanced metastasis in Panc02-3P cells were examined by in vitro experiments. First, the morphology of each cell derivative was microscopically observed. Parental Panc02 cells displayed a cobblestone-like shape with close cell–cell contact, whereas Panc02-3P cells lost cell–cell contact and exhibited a spindle shape (Fig. 2A). Moreover, phalloidin staining revealed an increased formation of actin stress fibers, an indicator of mesenchymal phenotype, in Panc02-3P cells (Fig. 2B). Thus, Panc02-3P cells underwent EMT after serial in vivo transplantations and acquired the mesenchymal phenotype. When we next assessed the proliferative capacity of the cells, Panc02-3P cells proliferated more rapidly than parental Panc02 cells in normal cell culture conditions (Fig. 2C). In addition, the migratory ability of Panc02-3P#1 cells increased, compared with that of parental Panc02 cells (Fig. 2D). These results suggested that both enhanced proliferation and increased invasive ability may contribute to the high metastatic ability of Panc02-3P cells.

### Gene signature(s) of highly metastatic Panc02-3P cells

**Fig. 3:**
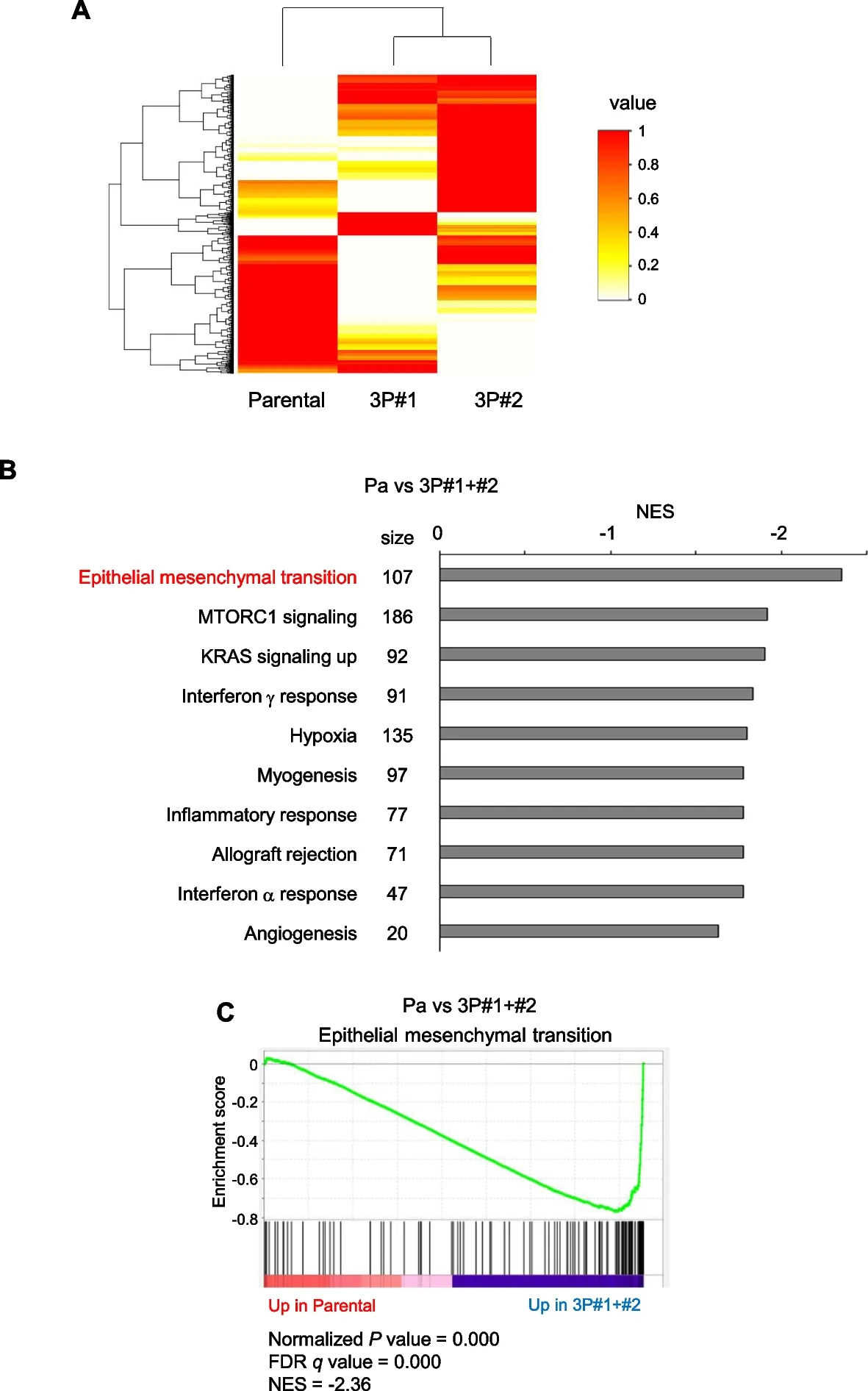
RNA-seq showing EMT induced in Panc02-3P cells. **A** Gene expression profiles using RNA-seq analysis. The cluster analysis is shown using a heatmap. Genes with an RPKM value of 3 or higher in at least one of the three cell lines were selected. Gene expression levels were subjected to min–max scaling so that the maximum and minimum values across the cell lines were set to 1 and 0, respectively, and a heatmap was generated. **B** The result of GSEA. Gene ontology analysis was performed using GSEA. The upregulated ontologies in Panc02-3P cells are shown. **C** Enrichment plot of EMT target genes in Panc02-3P cells. A gene set targeted EMT was identified as the most enriched phenotype

Gene expression of each derivative was profiled using RNA-seq analysis to identify the molecular mechanism(s) crucial for the highly invasive features of Panc02-3P cells. The expression profiles revealed a global transcriptomic shift between parental Panc02 cells and their highly metastatic derivatives, Panc02-3P cells (Fig. 3A). Elevated genes in Panc02-3P cells were subjected to GSEA. Gene sets related to EMT enriched in Panc02-3P cells, as expected from the results in Figs. 2 and 3B, C. In addition, following PCA demonstrated the different gene-expression patterns between parental Panc02 and Panc02-3P cells (Supplementary Fig. 1). Moreover, annotation analysis of top genes was consistent with acquired characteristics of malignant cells, including high tumorigenicity of cells and high metastatic ability of Panc02-3P cells. These results suggested that Panc02-3P cells acquired gene expression features associated with enhanced invasiveness, partially overlapping with EMT-related signatures, causing enhanced metastatic ability.

### Gene expression of EMT markers in Panc02-3P cells

**Fig. 4:**
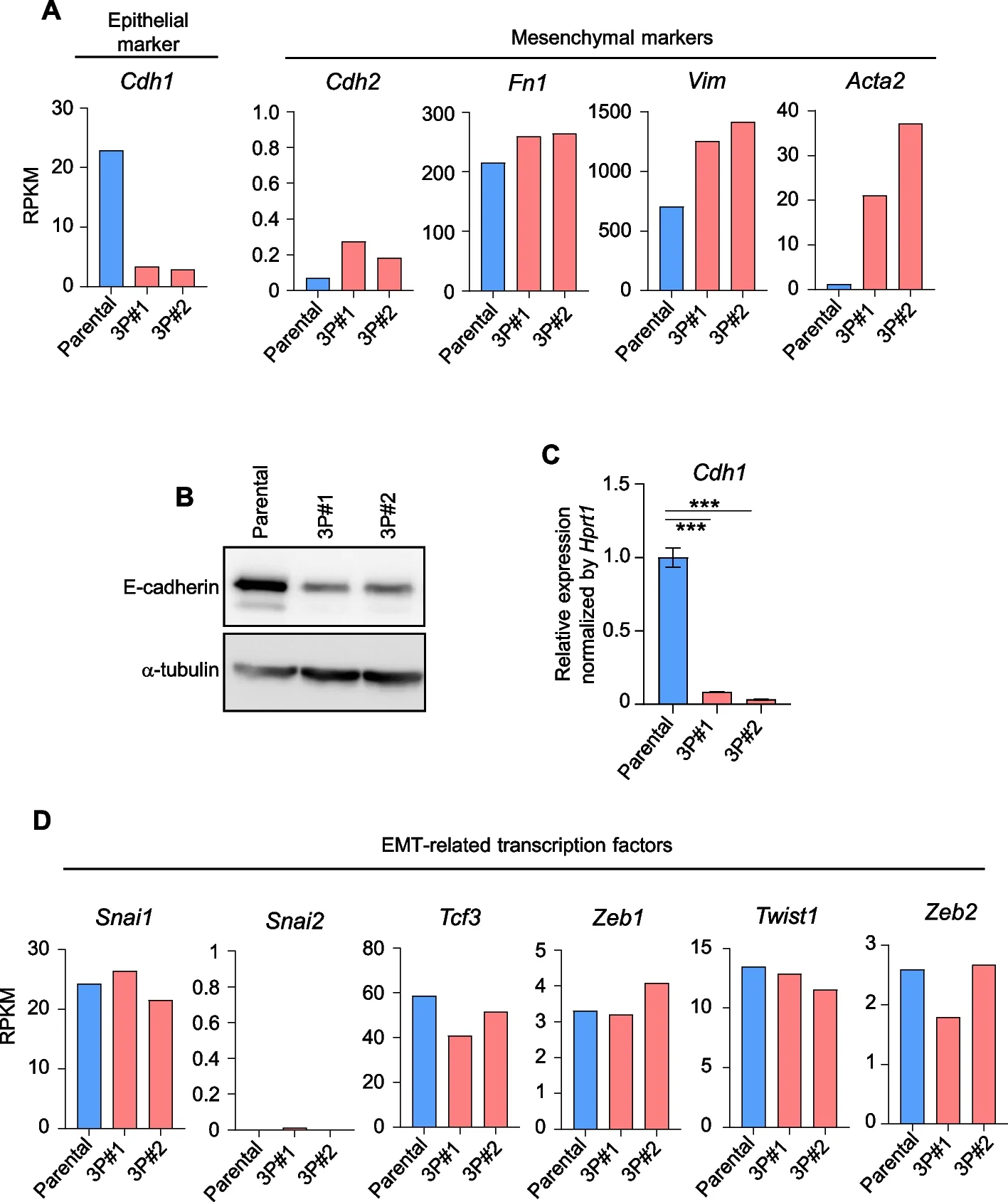
The expression of EMT-related genes in Panc02-3P cells. **A** Expression of epithelial or mesenchymal markers. The expression of *Cdh1*, *Cdh2*, *Fn1*, *Vim*, and *Acta2* in RNA-seq data is shown with RPKM. **B** Protein expression of E-cadherin was analyzed by immunoblotting. Representative data from independent experiments are shown. Cropped images of gels are shown; full-length images are provided in the Supplementary Fig. 4. **C** The mRNA expression of E-cadherin was studied by real-time PCR analysis. The expression was normalized by *Hprt1*. Data are presented as mean ± SD (*n* = 3). **D** Expression of EMT-TFs. From the result of RNA-seq analysis in (A), the expression of EMT-TFs (*Snai1*, *Snai2*, *Tcf3*, *Zeb1*, *Twist1*, and *Zeb2*) is shown with RPKM

In vitro experiments and RNA-seq indicated that Panc02-3P cells exhibited mesenchymal-like gene expression changes following serial transplantation. The RNA-seq results revealed that Panc02-3P cells demonstrated the downregulation of *Cdh1* (encoding E-cadherin) and upregulation of mesenchymal markers, including *Cdh2* (N-cadherin), *Fn1* (fibronectin), *Vim* (vimentin), and *Acta2* (α-smooth muscle; α-SMA) relative to parental Panc02 cells (Fig. 4A). As for E-cadherin, a typical epithelial marker, the downregulated expression was reproduced at both protein and mRNA levels (Fig. 4B and C). These data supported the hypothesis that Panc02-3P cells exhibited mesenchymal-like features, although canonical EMT transcriptional programs were not clearly activated. However, the expression of EMT-TFs *(Snail*, *Slug*, *Tcf3*, *Zeb1*, *Twist1*, and *Zeb2*) did not differ significantly between the derivatives (Fig. 4D). Considering this fact, we hypothesized that other factors may be involved in the acquisition of the mesenchymal phenotype of Panc02-3P cells.

### Identification of Lox family gene(s) upregulated in Panc02-3P cells

**Fig. 5:**
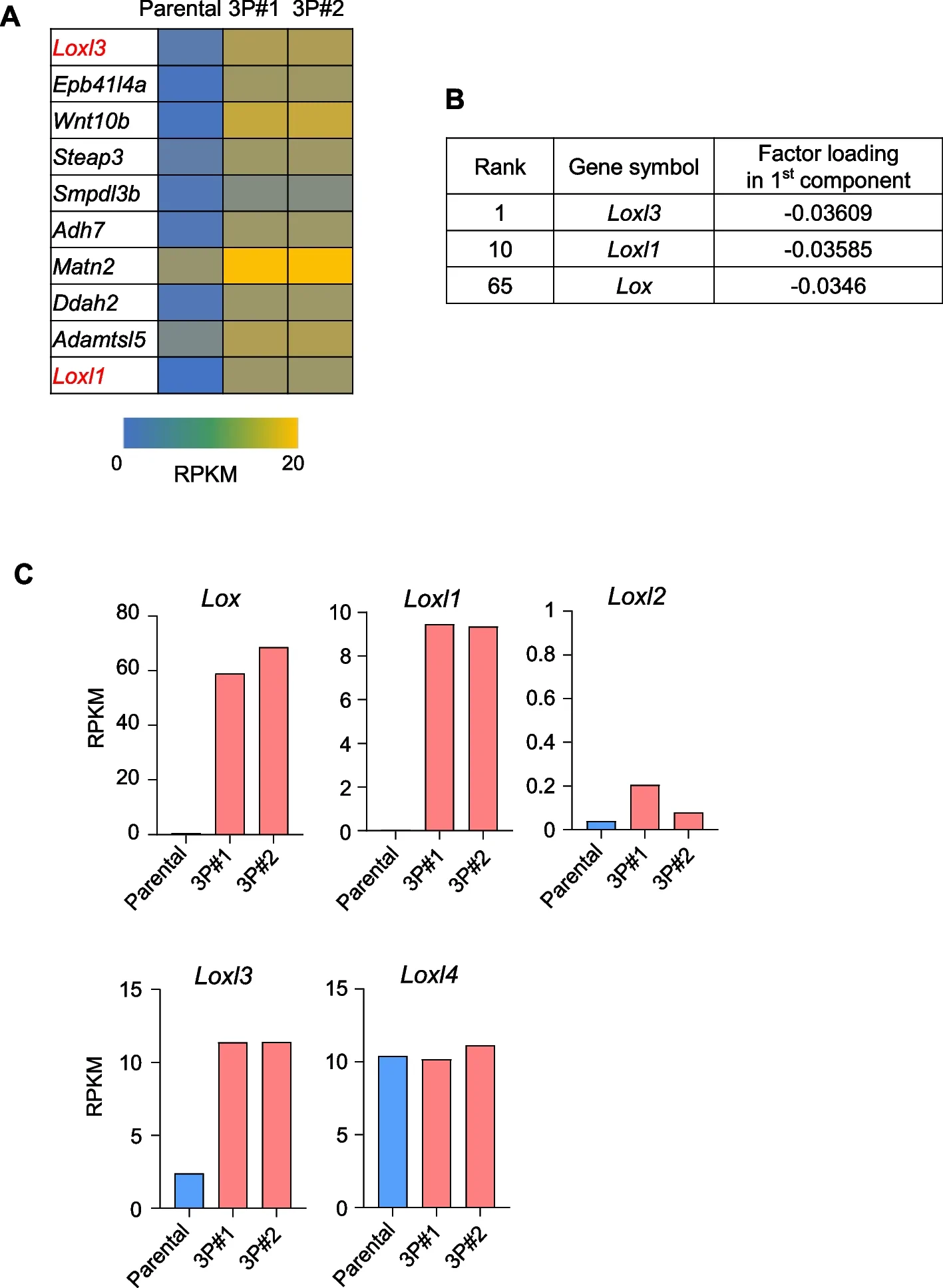
Expression of LOX family genes in Panc02-3P cells. **A** List of the top 10 genes upregulated in Panc02-3P cells. The result from RNA-seq analysis of parental Panc02, Panc02-3P#1, and 3P#2cells in Fig. 3A. **B** The result of RNA-seq analysis of the LOX family genes in Fig. 3A. The upregulation of the LOX family members is extracted from Supplementary Table S2. **C** Expression of the Lox family genes. From the result of RNA-seq analysis, the expression of LOX family genes (*LOX, LOXL1, LOXL2, LOXL3*, and *LOXL4*) is shown with RPKM

**Fig. 6:**
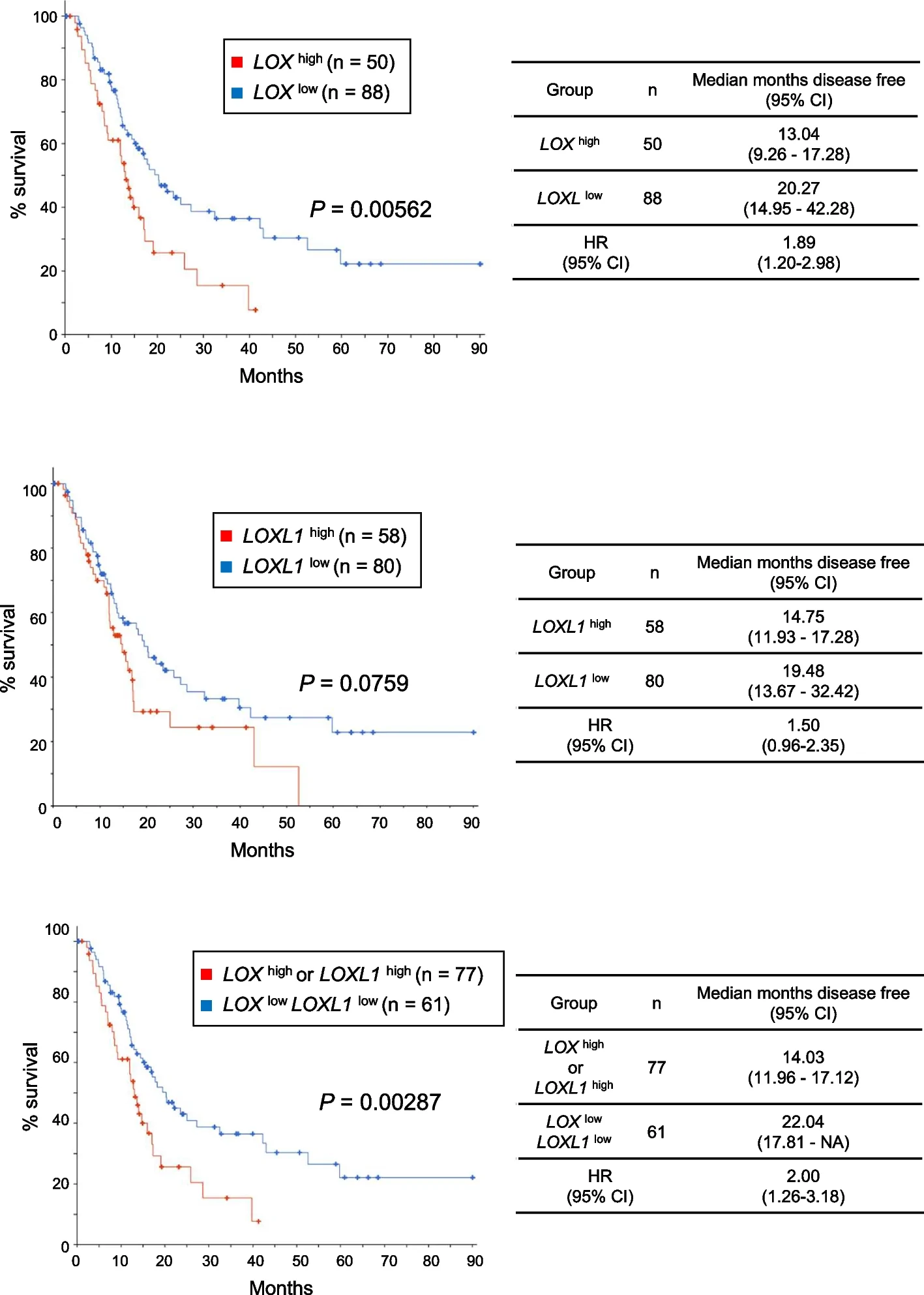
Survival rate of patients with pancreatic cancer. Correlation between LOXs expression and the disease-free survival of pancreatic adenocarcinoma patients. The TCGA database was analyzed and divided into high and low groups of each gene (log-rank test, *n* = 138 each). Kaplan–Meier plots (left) and disease-free median months (right) are indicated. mRNA expression levels of LOXs were standardized as z-scores, and patients were stratified into high- and low-expression groups using a cutoff of 0. Hazard ratios (HRs) were estimated using Cox proportional hazards regression

Focusing on the upregulated genes in Panc02-3P cells compared with the parental Panc02 cells, the expression of *Loxl3* and *Loxl1* was markedly increased (Fig. 5A, Supplementary Table 2), as well as *Lox* (Fig. 5A, B, C, and Supplementary Table 2). These results were confirmed by the independent real-time PCR analysis (Supplementary Fig. 2). Among the *Lox* family members, *Loxl3* was identified as the most highly upregulated gene in Panc02-3P cells (rank #1 in Fig. 5A), exhibiting approximately fourfold higher expression compared with the parental Panc02 cells. Given this prominent upregulation, *Loxl3* was initially considered a potential metastasis-associated candidate in our experimental model. However, analysis of publicly available pancreatic cancer patient datasets revealed that the association between *LOXL3* expression and patient prognosis was less pronounced (Supplementary Fig. 3). In contrast, the high expression of *LOX* is significantly associated with poor prognosis of pancreatic cancer patients (Fig. 6). Although statistical significance was not observed, *LOXL1* expression displayed a similar impact. Synchronously increased expression of *LOX* and *LOXL1* in patients with pancreatic cancer displayed the most significant difference in the survival rate (Fig. 6). These data suggested that the LOX family members, especially LOX and LOXL1, which are highly expressed in metastatic Panc02 derivatives, are intricately associated with the progression of pancreatic cancer.

### Role of Lox and Loxl1 in migration and invasive ability

**Fig. 7:**
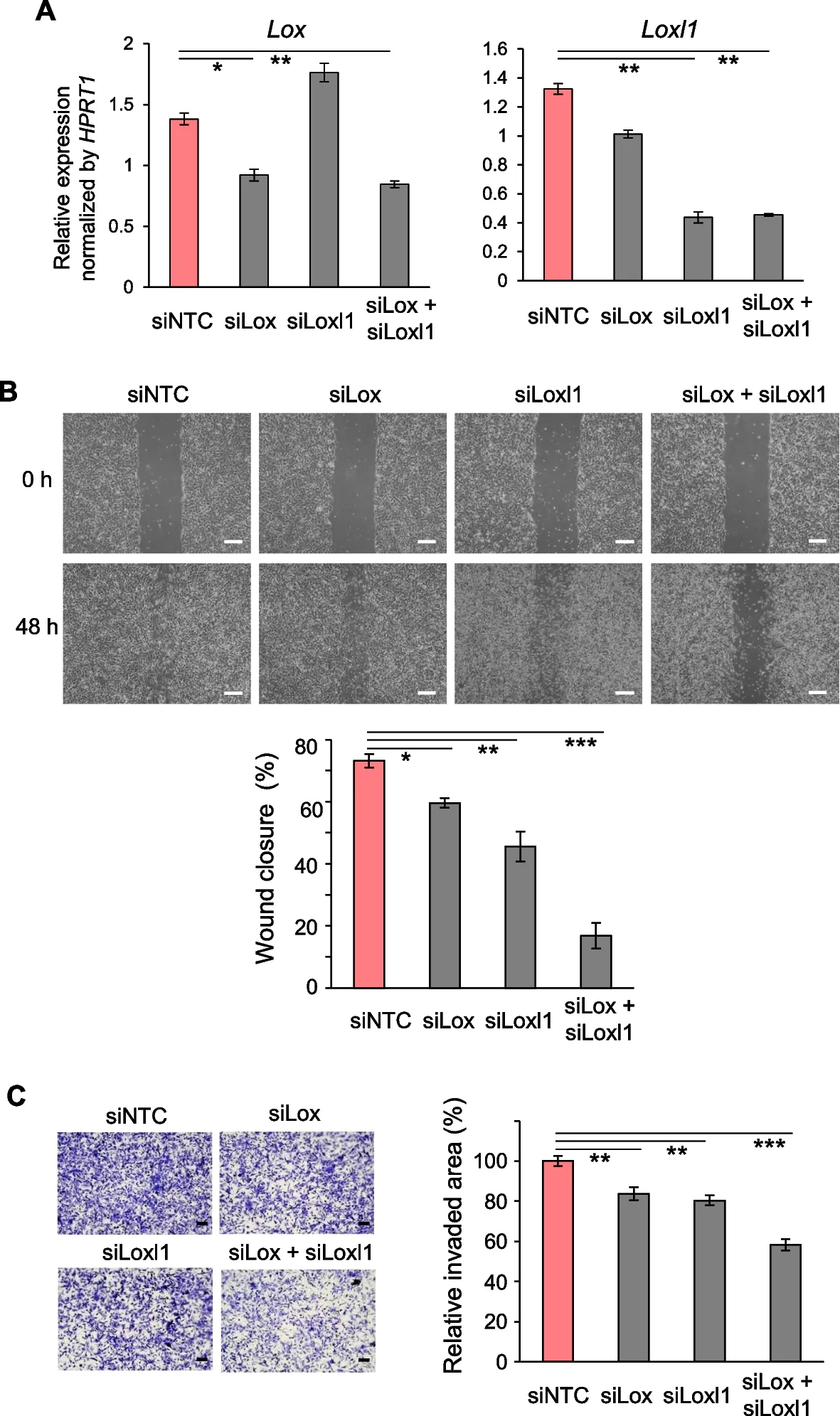
Effects of knockdown of LOX or/and LOXL1 expression in Panc02 derivatives. **A** The effect of siRNA on the expression of LOX and LOXL1. Panc02-3P#1 cells were treated with siRNA targeting LOX and LOXL1 (siLOX and siLOXL1). The expression was assessed by real-time PCR analysis. siNTC was used as a control. Data are presented as mean ± SD (*n* = 2). **B** The effect of siLOX or/and siLOXL1 on migration ability. Panc02-3P#1 cells were treated with siLOX or/and siLOXL1 and incubated for 24 h. Next, the wound was incised. Two days after the incision, the area of wounds was measured. Representative images (top) and quantification of the remaining wound area (bottom) are shown. Bar, 100 μm. Data are presented as mean ± SD (*n* = 2). **C** The effect of siLOX or/and siLOXL1 on invasive ability. Panc02-3P#1 cells were treated with siLOX or/and siLOXL1 and incubated for 24 h. Next, a transwell migration assay was conducted. The chambers were collected after another 24 h of incubation, and migrated cells were stained with Giemsa. Representative images (left) and quantification of the relative area covered by invaded cells (right) are shown. Bar, 100 μm. Data are presented as mean ± SD (*n* = 3). * P < 0.05. ** P < 0.01. *** *P* < 0.001

Migratory and invasive ability is crucial for metastasis, particularly during the intravasation step from primary tumors, and is also associated with cellular motility and invasive properties. We examined this property in our highly metastatic derivatives by assessing the migratory ability following siRNA-mediated knockdown. Transfection with siLOX and siLOXL1 effectively suppressed the expression of LOX and LOXL1 at the mRNA level, respectively (Fig. 7A). The result demonstrated that siLOX and/or siLOXL1 markedly reduced wound closure and migration compared with siNTC-treated cells (Fig. 7B and C). Moreover, the double knockdown of LOX and LOXL1 caused an even more pronounced reduction in migratory activity. These findings suggest that expression of LOX and LOXL1 is necessary for sustaining migration ability of Panc02-3P cells. Altogether, LOX and LOXL1 contribute to the migratory and invasive properties of pancreatic cancer cells. These data suggest a potential link between LOX/LOXL1 and the regulation of epithelial and mesenchymal features, including E-cadherin expression in pancreatic cancer cells.

## Discussion

The LOX family consists of amine oxidases, LOX and LOX-like 1–4 (LOXL1, LOXL2, LOXL3, and LOXL4), that catalyze the crosslinking of collagens and elastin, playing essential roles in ECM remodeling and stabilization [12]. Recent research has demonstrated tumor-promoting functions of LOX family members. The increased expression of LOX or LOXL2 has been reported in colorectal [25, 26], gastric [27], and breast cancer, and is correlated with poor prognosis [28, 29]. Increased LOXL2 expression in pancreatic cancer has been linked to EMT status and poor patient survival [30]. Consistent with these reports, we found a correlation between LOX/LOXL1 expression and unfavorable outcomes in pancreatic cancers. These findings indicate that targeting the LOX family may suppress malignant properties in pancreatic cancer.

In addition to their ECM-modifying roles, the members of the intracellular LOX family have been reported to exert cell-autonomous functions that directly affect cancer cell behavior [31, 32]. LOXL3 has been reported to be associated with EMT regulation [33] and as a factor sustaining genomic stability in human melanoma [34]. LOXL2 has been reported to promote EMT by downregulating the expression of E-cadherin by regulating Snail1 [30, 35, 36]. In our study, LOX, LOXL1, and LOXL3 were highly expressed in metastatic Panc02 derivatives. Silencing of *LOX* or *LOXL1* markedly reduced migration, and simultaneous knockdown of both genes further enhanced these effects. These findings support a tumor-promoting function of the LOX family in pancreatic cancer progression.

Among the LOX family members, our study is the first to demonstrate that LOX and LOXL1 function as modulators of migratory and invasive properties in pancreatic cancer cells. Collectively, this study highlights a novel mechanism by which the LOX family expression may contribute to the regulation of cancer cell behavior, including migratory and invasive phenotypes and pancreatic cancer progression. However, several questions remain. First, the mechanisms underlying the elevated LOX family expression in metastatic derivatives remain unclear. We have previously demonstrated that the pancreatic TME can “educate” cancer cells via epigenetic modifications, such as DNA methylation, thereby conferring malignant phenotypes [7, 37]. Consistently, LOX overexpression due to global hypomethylation promotes invasion and metastasis in esophageal cancer [38]. Given these findings, it is conceivable that the upregulation of the LOX family in our system results from TME-driven epigenetic regulation. Notably, we have previously conducted experiments using nude mice lacking a functional immune system [7, 37], whereas the present study employed immunocompetent mice. Therefore, the induction of the LOX family genes in this study might be influenced by interactions with the T-cell-dependent immune system, reflecting the impact of an intact immune microenvironment.

Second, although our results suggest that the LOX family members influence mesenchymal-like features independently of conventional EMT-TFs, the precise mechanism remains to be elucidated. Similar intracellular mechanisms may contribute to the regulation of migratory and invasive properties in pancreatic cancer cells. While some EMT-related gene expression changes were observed, our data do not fully support activation of a canonical EMT program, and therefore the contribution of EMT to this phenotype should be interpreted with caution. In addition, our preliminary data demonstrated that pan-LOX inhibitor β-aminopropionitrile (BAPN) did not significantly affect the migratory capacity and *CDH1* expression of Panc02-3P cells (unpublished data). These findings suggest that the pro-migratory effects of LOX family members may not be solely dependent on their canonical enzymatic activity. Moreover, while EMT has been associated with the acquisition of cancer stem cell-like traits, stemness-related phenotypes were not examined in Panc02-3P cells.

## Conclusion

Focusing on the role of TME is particularly significant in the study of PDAC, which is characterized by abundant ECM deposition and infiltration of different stromal and immune cells. Orthotopic inoculation of mouse models is widely used to reproduce pancreatic TME. We investigated the characteristics of highly metastatic Panc02 derivatives established from a syngeneic orthotopic inoculation model. These derivatives acquired an enhanced mesenchymal-like characteristics. In vitro culture of Panc02-3P cells is characterized by a spindle-shaped morphology, indicating stable and fixed mesenchymal features. Moreover, our findings suggest that their properties were sustained by epigenetic modifications, selections, or other mechanisms through serial transplantations. RNA-seq analysis identified the LOX family members as a key gene signature associated with metastatic phenotypes in these cells.

## Supplementary Information

Supplementary Material 1.

## Data Availability

The datasets during the current study are available from the corresponding author on reasonable request.
