# Supplementary material for: In vitro functional analysis of lysyl oxidase family members in highly metastatic pancreatic cancer cells derived from a syngeneic orthotopic model
Source: BMC Cancer. 2026 Jun 15;26:1015. doi: 10.1186/s12885-026-16173-1 (PMC13501788; doi:10.1186/s12885-026-16173-1)
Supplement: Supplementary file 1 [file 12885_2026_16173_MOESM1_ESM.docx]

**Supplementary Table 1 Mutations of driver genes in Panc02 cells.**

Mutations of representative pancreatic cancer driver genes in Panc02 cells are shown based on previous reports [1].

1. Wang Y, Zhang Y, Yang J, Ni X, Liu S, Li Z, Hodges SE, Fisher WE, Brunicardi FC, Gibbs RA, Gingras M-C, Li M. Genomic sequencing of key genes in mouse pancreatic cancer cells. Curr Mol Med. 2012;12:331–41.

**Supplementary Table 2 The gene list upregulated in Panc02 3P cells.**

The result of RNA-seq analysis in Fig. 3A. The top 75 genes in the first component are enlisted.

**Supplementary Fig. 1 Results of RNA-seq with PCA.**

The results of RNA-seq analysis in Fig. 3A were applied to PCA.

**Supplementary Fig. 2 Expression of Lox family genes in Panc02** **derivatives.**

The mRNA expression of the LOX family genes was examined in the Panc02 derivatives by real-time PCR analysis. The expression was normalized by that of *Hprt1*. Data are presented as mean ± SD (n = 2).

***

***

Parental

3P#1

3P#2

*Lox*

Relative expression normalized by *Hprt1*

(parental = 1)

0

100

150

50

Parental

3P#1

3P#2

*Loxl1*

0

1000

1500

500

Parental

3P#1

3P#2

*Loxl2*

0

1.5

2

0.5

1

Parental

3P#1

3P#2

*Loxl3*

Relative expression normalized by *Hprt1*

(parental = 1)

0

6

8

2

4

Parental

3P#1

3P#2

*Loxl4*

0

1

1.5

0.5

***

**

***

**Supplementary Fig. 3 Correlation between *LOX3* expression and the disease-free survival of**

**pancreatic adenocarcinoma patients.**

The TCGA database was analyzed and divided into high and low groups of *LOX3* gene (log-rank

test, n = 138). Kaplan-Meier plot is indicated.

**Supplementary Fig. 4. Uncropped full-length immunoblot images corresponding to Fig. 4B.**

Experiment #1 corresponds to the blot presented in Fig. 4B, whereas Experiment #2 represents an independent replicate experiment. Original chemiluminescence images, membrane images, and merged views are shown for E-cadherin (135 kDa) and α-tubulin (52 kDa), together with molecular weight markers and sample labels.
